# Ustekinumab trough levels predicting laboratory and endoscopic remission in patients with Crohn’s disease

**DOI:** 10.1186/s12876-022-02271-4

**Published:** 2022-04-21

**Authors:** Hisashi Hirayama, Yasuhiro Morita, Takayuki Imai, Kenichiro Takahashi, Atsushi Yoshida, Shigeki Bamba, Osamu Inatomi, Akira Andoh

**Affiliations:** 1 Department of Medicine, Shiga University of Medical Science, Seta-Tukinowa, Otsu, 520-2192 Japan; 2 Center for Gastroenterology and Inflammatory Bowel Disease, Ofuna Chuo Hospital, Kamakura, Japan

**Keywords:** Ustekinumab, Therapeutic drug monitoring, Enteroscopy

## Abstract

**Backgrounds:**

Optimal concentration of ustekinumab (UST) predicting endoscopic remission has not been fully investigated in Crohn’s disease (CD). We aimed to identify the optimal UST trough levels predicting clinical, laboratory and endoscopic remission in CD patients.

**Methods:**

Twenty-eight patients with CD were enrolled and investigated (27 patients by enteroscopy and 1 by colonoscopy). The endoscopic activity was assessed using the scoring system that applied the Rutgeerts score to observed intestine. Serum UST trough levels and anti-UST antibodies (AUAs) levels were determined by in-house immunoassays.

**Results:**

Endoscopic activity was negatively correlated with serum UST trough levels (Spearman’s rank correlation coefficient (ρ) = − 0.66, *P* = 0.0001) and serum albumin levels (ρ = − 0.60, *P* = 0.0007). The endoscopic activity was positively and significantly correlated with CRP (ρ = 0.59, *P* = 0.0009) and ESR (ρ = 0.44, *P* = 0.033). There was no significant association between the endoscopic score and AUA levels and/or Crohn’s disease activity index (CDAI). Serum UST trough levels and albumin levels were significantly higher in the endoscopic remission group (scores of 0 and 1) than in the non-endoscopic remission group (UST trough, mean 3.3 vs. 1.8 μg/mL). No significant difference was observed in AUAs between the endoscopic remission and non-remission groups. Receiver operation curve (ROC) analysis revealed that the optimal cutoff value of UST trough levels predicting normal CRP and serum albumin levels was 1.7 μg/mL for each, and the optimal cutoff value predicting endoscopic remission was 2.0 μg/mL (AUC: 0.80, 95% CI 0.64–0.96).

**Conclusion:**

Achievement of endoscopic remission requires higher UST trough levels than required for normalization of CRP and serum albumin levels.

## Introduction

Crohn’s disease (CD) is an inflammatory bowel disease (IBD) characterized by clinical symptoms such as abdominal pain, chronic diarrhea, gastrointestinal bleeding and intestinal complications such as strictures and fistulas [1, 2]. While the precise pathogenesis of CD remains unclear, it is believed to be caused by a combination of environmental, immune, and microbial factors in genetically susceptible individuals [1, 2]. There is currently no cure for CD, and the main purpose of treatment is achieving long-term remission to prevent irreversible gastrointestinal damage and disability [3, 4]. The treatment consists of immunomodulating drugs, such as corticosteroids, immunosuppressants and biologics [4]. Of these, the use of biologics such as anti-TNFα drugs has revolutionized the treatment of CD [5].

Interleukin (IL)-12 and IL-23 play crucial roles in the pathogenesis of IBD through induction of T-helper (Th)1 and Th17 responses [6, 7]. Ustekinumab (UST) is a human immunoglobulin (Ig)G1 monoclonal antibody targeting the p40 subunit of human IL-12/IL-23 and blocks Th1 and Th17 responses involved in the pathophysiology of CD [8]. Previous studies have shown that UST is effective for the induction and maintenance of clinical remission in patients with moderate to severe CD and ulcerative colitis [9–14]. Recently, Sandborn et al. reported that UST maintained a high clinical remission rate for 5 years without new safety signals in patients with CD [15].

The number of reports on the therapeutic drug monitoring (TDM) of UST in IBD patients are increasing [15–22] but an optimal concentration of UST predicting endoscopic remission has not been fully elucidated in CD patients. TDM offers a guide for selecting the best therapeutic option in the event of a patient losing their response to treatment. We have previously reported new immunoassays for the measurement of serum UST and anti-UST antibody (AUA) concentrations. These assays are low cost and need no special materials such as radioisotope and/or anti-UST idiotype antibodies and no expensive measurement devices. In this study, we investigated the optimal UST trough levels predicting clinical, laboratory and endoscopic remission in CD patients.

## Materials and methods

### Patients

Twenty-eight moderate to severe active patients with CD were enrolled from September 2017 to August 2020. These patients were treated with UST at the Shiga University of Medical Science Hospital. The demographic characteristics of the patients are described in Table 1. Clinical disease activity was evaluated using the Crohn’s disease activity index (CDAI) score [23]. Median CDAI was 210. Patients without active endoscopic disease and age ≤ 16 were excluded. Similarly, patients with a diagnosis of IBD unclassified, as well CD patients with pure perianal involvement without luminal disease, were excluded.

**Table 1:** Patient characteristics

Age, median (range)	37 (21–72)
Female/Male	10/18
*Montreal classification of Crohn’s disease*	
Location, n (%)	
L1 Ileal	9 (32)
L2 Colonic	2 (7)
L3 Ileocolic	17 (61)
Behavior, n (%)	
B1 non-stricturing, non-penetrating	5 (18)
B2 stricturing	21 (75)
B3 penetrating	2 (7)
Medication, n (%)	
5-ASA	21 (75)
Azathioprine	14 (50)
Prednisolone	4 (14)
Biologics naïve, n (%)	12 (32)
Duration of UST treatment (weeks), median (range)	48 (24–112)
Endoscopic examination (weeks), median (range)	49 (18–112)

*5-ASA*, 5-aminosalicylic acid; *UST*, ustekinumab

UST was introduced by a one-time intravenous infusion according to the patient’s body weight (260 mg for patients < 55 kg, 390 mg for patients between 55 and 85 kg, and 520 mg for patients > 85 kg). The patients then received a UST subcutaneous injection (90 mg/body) every 8 weeks. Blood was collected before the next injection (trough concentration). There was an average of 6.5 UST injections at the time of endoscopy.

### Ethics

The study protocol was approved by the institutional review boards of the Shiga University of Medical Science (permission No. R2017-136). All patients gave their written informed consent prior to their inclusion in this study. The registration number of the University Hospital Medical Information Network Center (UMIN) was 000033552.

### Endoscopic examination

Trans-anal approach using the single-balloon enteroscope Olympus SIF-Q260 (Olympus, Tokyo Japan) was performed in all patients except those with a stoma. For a patient with a stoma, the colonoscope Olympus PCF-Q260 (Olympus) was used.

The trans-oral approach was applied if jejunal lesions were suspected by other diagnostic modalities, i.e., small bowel follow-through, computed tomography, and/or magnetic resonance enteroclysis.

### Evaluation of small bowel lesions

Endoscopic activity of CD was assessed using the scoring system as described in our previous report [24] (Table 2). The original Rutgeerts score [25] was developed for evaluation of anastomosis lesions after ileocolic resection, but in this study we adapted it for entire endoscopically observed lesions. The score of the most serious lesion was adopted. Endoscopic remission was defined as a score of 0 (no lesions or scar) or 1 (≤ 5 aphthous lesions). At least two well-trained endoscopists calculated the disease scores in patients. Endoscopic evaluation was performed within 1 weeks before and after UST injection.

**Table 2:** Endoscopic scores after ustekinumab treatment

Score	Definition	n	Total
0	No lesions or scar	6	8 (28.6%)
1	≤ 5 aphthous lesions	2	
2	> 5 aphthous lesions with normal mucosa between the lesions	5	20 (71.4%)
3	Diffuse aphthous lesions including smaller ulcers (0.5–2 cm in diameter)	8	
4	Diffuse inflammation with larger ulcers (> 2 cm in diameter)	7	

We used the endoscopic scoring system as described in our previous report [24]. The original Rutgeerts score [25] was developed for the evaluation of anastomosis lesions after ileocolic resection, but in this study we adapted it for entire endoscopic lesions. The score of the most serious lesion was adopted. Endoscopic remission was defined as a score of 0 (no lesions or scar) or 1 (≤ 5 aphthous lesions)

### Measurement of serum UST concentrations

Serum UST levels were determined by an immunoassay developed in our laboratory [26]. Briefly, an avidin ELISA plate® (blocking-less type; Sumitomo Bakelite Co., Ltd., Tokyo, Japan) was coated with biotinylated-IL-12 p40 (100 μl of 0.5 μg/mL) by incubation for 2 h. After extensive washing, a further blocking was performed with Block Ace® (DS Pharma Biomedical, Co., Ltd., Suita, Japan). After washing, samples (100 μL of 100-fold diluted serum) were incubated overnight at 4 °C. Finally, the reacted UST was detected by horseradish peroxidase -labeled F(ab′)_2_ fragments of chicken anti-human IgG (× 20,000 diluted; Thermo Fisher Scientific Co., Ltd., Waltham, MA). 3,3′,5,5′-Tetramethylbenzidine (Nacalai Tesque, Kyoto, Japan) was used for color development.

### Measurement of serum AUA concentrations

Serum levels of anti-UST antibodies (AUAs) were measured using a drug-tolerant assay developed in our laboratory [26]. Briefly, immune complexes of ustekinumab and AUA in samples were dissociated by treatment with 0.1 M glycine–HCl buffer (pH 2.7) and IgG fraction was isolated using protein G beads. IgG was eluted and the concentration was adjusted to 20 μg/ml IgG with a carbonate-bicarbonate buffer (pH 9.6). Each well of a 96-well ELISA plate was coated with diluted IgG containing AUAs (100 μl) overnight. AUAs on the plate were detected by 3 h incubation with HRP-labeled ustekinumab (100 μl of 2.0 μg/ml). 3,3′,5,5′-Tetramethylbenzidine was used for color development. The values were reported in μg/ml-calibrated (μg/ml-c) according to calibration standards using polyclonal goat anti-human IgG (MP Biomedicals, LLC, Solon, OH).

### Statistical analyses

The Chi-square or Mann–Whitney U test was used to evaluate the difference between two independent groups. The Spearman’s rank correlation coefficient was used to evaluate associations between parameters. The cut-off values of UST concentration associated with normal C-reactive protein (CRP), serum albumin and endoscopic remission were determined using receiver operating characteristic (ROC) curve analysis. All statistical testing was performed at the 0.05 significance level.

## Results

**Fig. 1:**
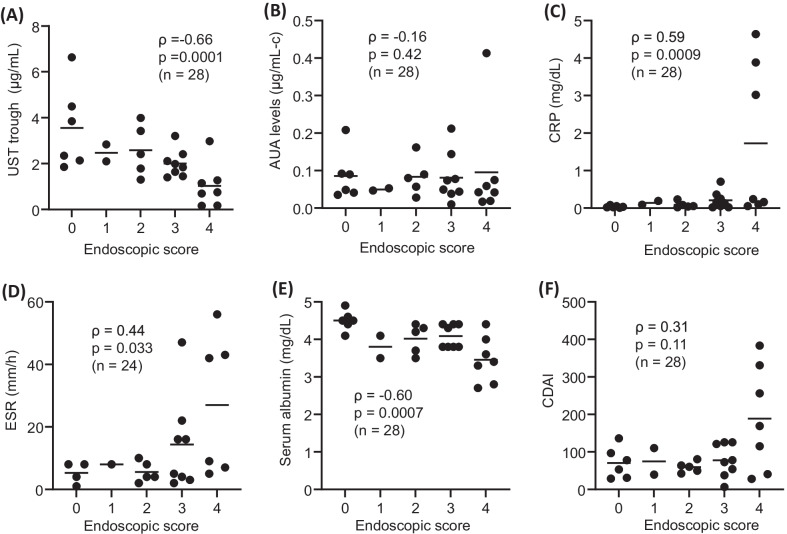
Association between the endoscopic score and laboratory and clinical parameters. Endoscopic activity of CD was assessed using the scoring described previously [24] (Table 2). The original Rutgeerts score [25] was developed for evaluation of anastomosis lesions after ileocolic resection, but we adapted it for the most serious lesion of the entire region observed by enteroscope. The Spearman’s rank correlation coefficient (ρ) for non-parametric correlations is presented

Based on endoscopic findings, we initially evaluated the relationship between the endoscopic score and serum UST trough levels as well as AUA, CRP, ESR, serum albumin and CDAI. As shown in Fig. 1A, a significant, negative correlation between the endoscopic activity and serum UST trough levels was observed (Spearman’s rank correlation coefficient (ρ) = − 0.66, *P* = 0.0001). A similar negative correlation was detected between the endoscopic activity and serum albumin levels (ρ = − 0.60, *P* = 0.0007) (Fig. 1E). The endoscopic score was positively and significantly correlated with CRP (ρ = 0.59, *P* = 0.0009; Fig. 1C) and ESR (ρ = 0.44, *P* = 0.033; Fig. 1D). However, there was no significant association between the endoscopic score and AUA levels (ρ = − 0.16, *P* = 0.42; Fig. 1B) and/or CDAI (ρ = 0.31, *P* = 0.11; Fig. 1F).

**Fig. 2:**
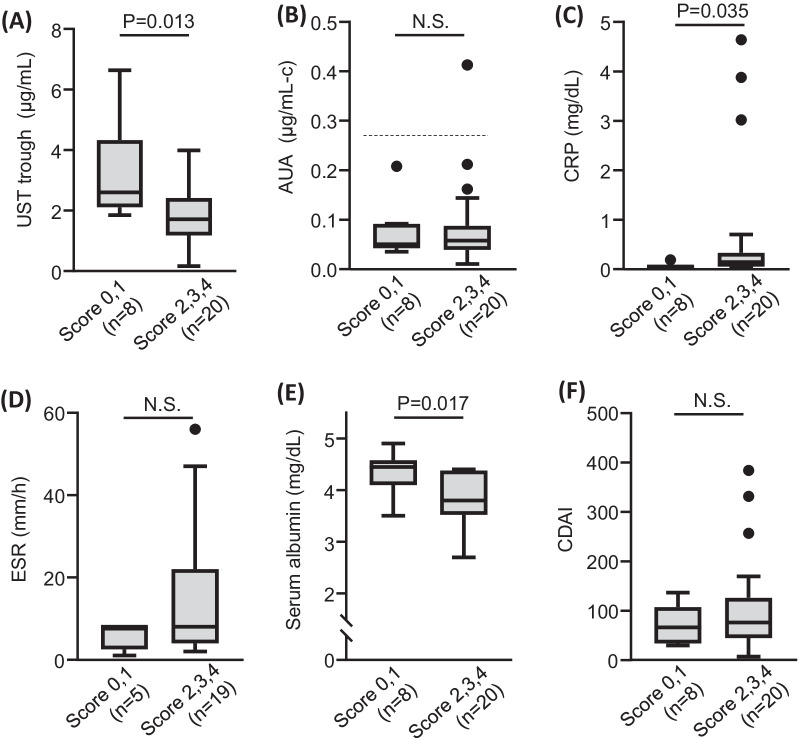
Comparison of clinical markers between patients with endoscopic remission (score 0 and 1) and patients with no endoscopic remission (score 2, 3 and 4). The dashed line in AUA indicates the cutoff value to be judged as positive (0.27) [26]. UST, ustekinumab; AUA, anti-ustekinumab antibodies; CDAI, Crohn’s disease activity index

Endoscopic remission was achieved in 8 of 28 patients (28.6%) (Table 2). As shown in Fig. 2, serum UST trough levels and albumin levels were significantly higher in the endoscopic remission group (scores of 0 and 1) than in the non-endoscopic remission group (scores of 2, 3, 4) [UST trough, mean 3.3 μg/mL (remission) vs. 1.8 (non-remission); serum albumin, 4.3 vs. 3.9 mg/dL)] (Fig. 2A and E). In contrast, CRP levels were significantly higher in the non-mucosal healing group compared to the mucosal healing group (0.71 vs. 0.06 mg/dL) (Fig. 2C). No significant differences were observed in AUAs, ESR and CDAI between the mucosal healing group and the non-mucosal healing group. The cutoff value of AUA was 0.27 μg/mL-c [26] and only one patient of the non-endoscopic group was positive (3.5%).

**Fig. 3:**
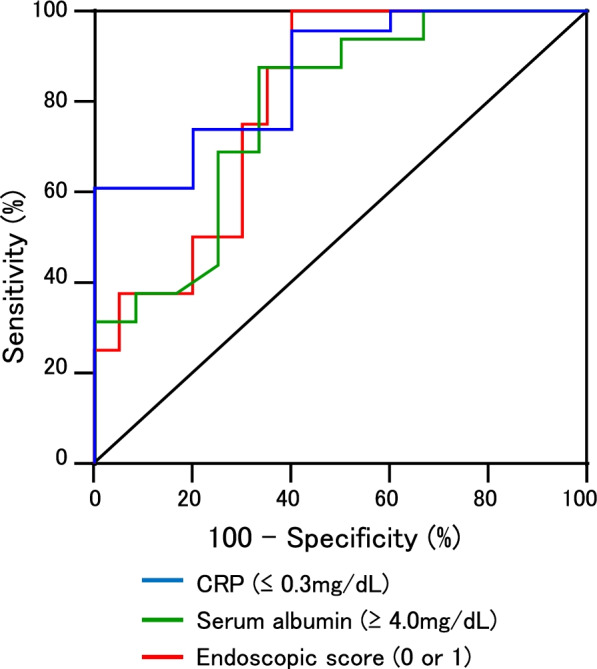
Receiver operation curve (ROC) of ustekinumab trough levels for identification of normal CRP levels (≤ 3.0 mg/dL), albumin levels (≥ 4.0 mg/dL), and mucosal healing (endoscopic score 0 and 1). The results of statistical analyses were presented in Table 3

The power of serum UST trough levels to predict normal clinical laboratory data and endoscopic remission (scores of 0 and 1) was evaluated. As shown in Table 3 and Fig. 3, the accuracy of UST trough levels for identification of patients with normal CRP levels (≤ 0.3 mg/dL) was sufficient (area under the curve [AUC]: 0.86, 95% CI 0.70–1.00). The most accurate cutoff value predicting normal CRP levels was 1.7 μg/mL. Similar results were observed in identification of normal albumin levels (≥ 4.0 mg/dL). The optimal cutoff value predicting normal albumin levels was a UST trough of 1.7 μg/mL. In addition, identification of endoscopic remission (scores of 0 and 1) using UST trough levels required a higher cutoff value of 2.0 μg/mL (AUC: 0.80, 95% CI 0.64–0.96).

**Table 3:** Predictive ustekinumab trough levels for laboratory and endoscopic remission

	CRP (≤ 0.3 mg/dL)	Serum albumin (≥ 4.0 mg/dL)	Endoscopic score (0 or 1)
n (yes/no)	23/5	16/12	8/20
AUC mean (95% CI)	0.86 (0.70–1.00)	0.78 (0.61–0.96)	0.80 (0.64–0.96)
*P* value	0.013	0.011	0.015
OR (95% CI)	7.9 (1.2–50.7)	3.2 (1.1–9.2)	2.6 (1.1–6.5)
Sensitivity	73.9	87.5	87.5
Specificity	80	66.7	65
UST trough cutoff (μg/mL)	1.71	1.71	2.04

*CRP*, C-reactive protein; *AUC*, area under the curve; *UST*, ustekinumab; *OR*, odds ratio

**Fig. 4:**
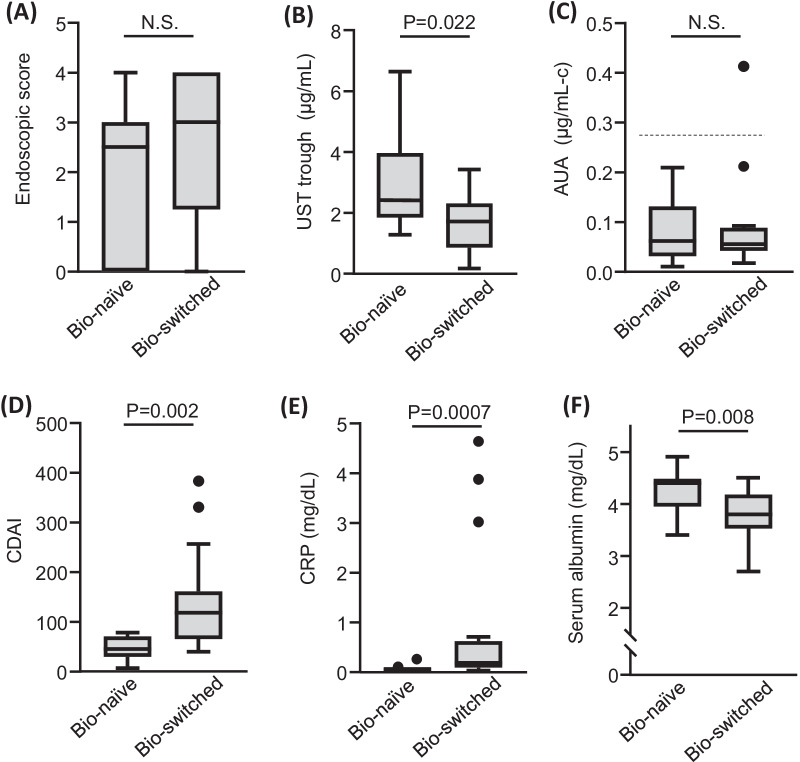
Comparison of endoscopic and clinical markers between bio-naïve patients (n = 12) and bio-switched patients (n = 16). The dashed line in AUA indicates the cutoff value to be judged as positive (0.27) [26]. UST, ustekinumab; AUA, anti-ustekinumab antibodies; CDAI, Crohn’s disease activity index

Previous studies have demonstrated that better clinical and endoscopic responses to biologics can be expected in biologics-naïve patients compared to biologics-switched patients [11, 17]. Although there was no difference in the endoscopic score between the biologics-switched and biologics-naïve patients (mean 2.5 vs. 2.0, *P* = 0.40) (Fig. 4A), CDAI and CRP were significantly higher and serum UST trough and albumin levels were significantly lower in the biologics-switched patients than in the biologics-naïve patients (Fig. 4B–F).

## Discussion

We investigated the association of the endoscopic disease activity with various parameters including UST trough levels and AUA levels in CD patients on UST maintenance therapy. The aggravation of the endoscopic activity was significantly associated with lower UST trough levels and serum albumin levels. The endoscopic score was positively and significantly correlated with CRP and ESR but not with AUA levels or CDAI score. UST trough levels were significantly higher in the endoscopic remission group than the non-endoscopic remission group. The optimal cutoff levels of UST for predicting normal CRP and/or serum albumin levels was 1.7 μg/mL, and achievement of endoscopic remission required a higher cutoff value of 2.0 μg/mL.

The evaluation of small bowel lesions is important but relatively difficult in the clinical setting of CD. Previous studies have used the Crohn’s Disease Endoscopic Index of Severity (CDEIS) [27] and/or the Simple Endoscopic Scores for Crohn’s Disease (SES-CD) [28]. However, these scores mainly focus on colonic lesions and are somewhat weak for evaluation of small-bowel lesions and definition of endoscopic remission by these scores is quite difficult. We previously introduced the Rutgeerts scoring system for the assessment of mucosal response to infliximab [24]. This scoring system evaluates the most serious lesion within an endoscopically-observed area according to the original Rutgeerts scoring system [25]. A score of 0 (no lesions or scar) or 1 (≤ 5 aphthous lesions) of the most serious lesions was considered to be indicative of endoscopic remission. This does not reflect total disease activity such as the extent of inflammation, but an evaluation of the endoscopically most serious lesion was acceptable as one of the appropriate parameters for evaluation of endoscopic remission.

Endoscopic response is established as a surrogate marker for effective control of CD that predicts a better outcome of the disease [29–31]. However, there are a limited number of reports on the association of UST trough levels with endoscopic response in CD patients [32]. As for the findings in the IM-UNITI maintenance study (mix of 8- or 12-week injections) [17], Adedokun et al*.* reported that the endoscopic remission rate at week 44 was significantly higher in patients with a UST trough > 0.5 μg/mL than those with ≤ 0.5 μg/mL, and that the proportion of patients with endoscopic remission reached a plateau at a UST level of 0.5 > to ≤ 1.4 μg/mL. They assessed the association between endoscopic remission rate and UST trough levels but did not determine a cutoff value predicting endoscopic remission. Battat et al*.* administered UST every 4 weeks as opposed to the standard regimens of 8- or 12-week intervals and reported that an optimal UST trough level predicting endoscopic response at week 26 was 4.5 μg/mL [20]. The short interval between injections might have accounted for this high trough result. A recent study by Takenaka et al*.* reported that achievement of endoscopic remission in the small bowel needs higher trough levels of biologics than that in the colon [30] and that the patients with UST levels of 4 µg/mL were 14.7-times more likely to exhibit endoscopic remission of the small bowel [30]. We showed that achievement of endoscopic remission required 2.0 μg/mL of UST trough levels in CD patients receiving 8-week interval injections. Our result seems to be relatively lower than the results of Takenaka et al*.* [30]. Various factors account for the results of pharmacokinetic study of UST, such as distinct treatment regimens (8- or 12-week intervals), disease outcome assessment, different assays for UST measurement and distinct patients’ backgrounds (e.g., biologics naïve or not). Some of these factors such as a strict endoscopic survey of the entire small bowel in Takenaka’s study might account for the discrepancy between the findings of their report and the current study. Furthermore, in our study only a few patients showed 4 μg/mL UST trough levels suggesting that the use of different assay systems might have influenced the results of both studies.

UST trough levels were significantly higher in the endoscopic remission group than the non-endoscopic remission group. One of the important factors affecting UST levels is an appearance of anti-drug antibodies. However, the involvement of this mechanism is unlikely, since we have previously reported a low immunogenicity of UST (positive rate approximately 7%) using a simple drug-tolerant assay developed in our laboratory [26]. In this study, only one of 28 patients (2.8%) showed a positive result. The absence of effects of AUAs on UST trough levels in this study is supported by the finding of no association of endoscopic activity and AUA levels or no differences in AUA levels between the endoscopic remission and non-endoscopic remission groups.

One of the mechanisms contributing to UST trough levels is an increased consumption of UST by active inflammation in the mucosa. Increased generation of cytokines including IL-12/23 consumes more UST at the active lesions of CD and may lead to a lower UST trough level. This may be supported by the finding that without an elevation of AUA levels, UST trough levels were significantly lower in the biologics-switched patients who showed a significant elevation of CDAI and CRP levels. This may also be supported by a recent study where dose intensification of UST therapy (4- or 6-week interval injections) was effective for CD patients who experienced a loss of response to UST under standard maintenance therapy [33].

This study includes several limitations. First, it is retrospective in design, which may lead to an increased risk of selection bias. Second, the backgrounds of patients such as duration of UST treatment and types of anti-TNF drugs and exposure duration were not consistent. Finally, our analysis was performed in two centers and limited by the sample size, and subsequent studies with larger cohorts are necessary to confirm our findings.

In conclusion, we demonstrated a relationship between serum UST trough levels and the endoscopic disease activity of CD patients on UST maintenance therapy. It is clear that achievement of endoscopic healing requires higher UST trough levels than those needed to achieve normalization of other laboratory parameters. The measurement of UST trough levels combined with other biomarkers might help to determine a therapeutic strategy for achieving endoscopic remission. Further prospective studies should be conducted to confirm the importance of measuring UST trough levels for predicting the endoscopic outcome of UST maintenance therapy.

## Data Availability

The datasets used during the current study are available from the corresponding author on reasonable request.

## Abbreviations

UST: Ustekinumab
AUA: Anti-ustekinumab antibody
IBD: Inflammatory bowel disease
CD: Crohn’s disease
UC: Ulcerative colitis
ESR: Erythrocyte sedimentation ratio
CRP: C-reactive protein
CDAI: Crohn’s disease activity index
AUC: Area under the curve
ROC: Receiver operating characteristic
Th: T-helper
Ig: Immunoglobulin
TDM: Therapeutic drug monitoring
